# Differential Changes with Age in Multiscale Entropy of Electromyography Signals from Leg Muscles during Treadmill Walking

**DOI:** 10.1371/journal.pone.0162034

**Published:** 2016-08-29

**Authors:** Hyun Gu Kang, Jonathan B. Dingwell

**Affiliations:** 1 Kinesiology, California State University San Marcos, San Marcos, California, United States of America; 2 Kinesiology and Health Education, University of Texas at Austin, Austin, Texas, United States of America; 3 Biomedical Engineering, University of Texas at Austin, Austin, Texas, United States of America; Duke University, UNITED STATES

## Abstract

Age-related gait changes may be due to the loss of complexity in the neuromuscular system. This theory is disputed due to inconsistent results from single-scale analyses. Also, behavioral adaptations may confound these changes. We examined whether EMG dynamics during gait is less complex in older adults over a range of timescales using the multiscale entropy method, and whether slower walking attenuates this effect. Surface EMG was measured from the *left vastus lateralis* (VL), *biceps femoris* (BF), *gastrocnemius* (GA), and *tibialis anterior* (TA) in 17 young and 18 older adults as they walked on a treadmill for 5 minutes at 0.8x-1.2x of preferred speed. Sample entropy (SE) and the complexity index (C_I_) of the EMG signals were calculated after successive coarse-graining to extract dynamics at timescales of 27 to 270 Hz, with *m* = 2 and *r* = 0.15 SD. SE and C_I_ were lower across the timescales in older adults in VL and BF, but higher in GA (all p<0.001); these results held for VL and GA even after accounting for longer EMG burst durations in older adults. C_I_ was higher during slower walking speed in VL and BF (p<0.001). Results were mostly similar for *m* = 3 and *r* = 0.01–0.35. Smaller *r* was more sensitive to age-related differences. The decrease in complexity with aging in the timescales studied was limited to proximal muscles, particularly VL. The increase in GA may be driven by other factors. Walking slower may reflect a behavioral adaptation that allows the nervous system to function with greater complexity.

## Introduction

Common gait alterations with aging include slower walking speed [1], increased kinematic variability [2], sensitivity to local perturbations [3], and decreased ankle push off [4]. However, the mechanisms for these behavioral alterations are not clear. These behavioral changes in aging have been hypothesized to be a result of a multi-system dysregulation [5], where interactions between the physiological systems that influence behavior break down, and thus limit function. Some alterations, such as walking slower, may instead be adaptive responses to attenuate the effects of such dysregulation.

The changes in physiological dynamics due to aging and disease have been observed in cell membranes [6], the cardiovascular system [7], postural control [8] and gait [9] where the observed dynamics decrease in complexity. Here, complexity is defined as the presence of irregular dynamics over a wide range of time or spatial scales of a physiological variable, as quantified using various entropy metrics.

Previous studies of aging motor function, however, have been inconclusive with regards to this question. Various single-scale methods were used to quantify dynamics at a particular timescale [10–14], but each method studied behavior at a different timescale, with different results. Such heterogeneity in methodology has made the synthesis across these results difficult. As these studies indicate, physiological systems appear to exhibit different types of behavior at different timescales. Therefore, studying motor behavior over a range of timescales would allow for a more complete picture.

Therefore, our purpose was to more completely describe neuromuscular behavioral changes in gait due to aging, by quantifying the effect of age on the complexity of muscle activation patterns during walking as measured using the multiscale entropy method on surface electromyography (EMG) signals. We tested whether: (1) older adults exhibit less complex dynamics as predicted by the multisystem dysregulation hypothesis once we consider a range of timescales; and (2) these effects are less present when walking slower, possibly attenuating such effects in older adults who may exhibit slow gait.

## Methods

### Subjects

Eighteen healthy older adults (age 65–85 years) and eighteen height-, weight-, and gender-matched young adults (age 18–28 years), participated after providing written informed consent as approved by the University of Texas at Austin institutional review board (Table 1). Data from one young adult was discarded due to poor quality, resulting in data from 17 young adults. We excluded those who reported recent lower extremity injuries, visible gait asymmetries or disabilities, or taking medications that may influence gait.

**Table 1:** Subject Characteristics [15].

	Young adults	Older Adults	p-value
Gender (M/F)	12/5*	12/6	0.55**
Age (years)	23.3 ± 2.6	72.1 ± 6.0	<0.0001
Height (m)	1.73 ± 0.094	1.70 ± 0.104	0.36
Body Mass (kg)	71.1 ± 9.86	73.2 ± 12.3	0.58
Preferred Walking Speed (PWS) (m/s)	1.30 ± 0.10	1.29 ± 0.15	0.86
PWS Range (m/s)	1.16–1.56	0.93–1.52	

* Reflects the number after the data for one young subject were discarded

**Fisher’s Exact Test (χ^2^).

Subjects walked on a treadmill (Desmo S model, Woodway USA, Waukesha WI) while wearing a safety harness (Protecta International, Houston TX). Each subject’s preferred walking speed (PWS) was determined, which allowed for treadmill acclimation. Subjects completed two 5-minute walking trials each at 5 different speeds (0.8, 0.9, 1.0, 1.1, 1.2×PWS), and with in-between rests of 2 minutes. The order of presentation was randomized while preventing two consecutive fast speed trials to avoid fatigue. Subjects were instructed to look straight ahead and avoid extraneous movements. Kinematics were measured using Vicon 612 (Oxford Metrics, UK). Other details of data collection and standard EMG analyses are presented elsewhere [15].

Muscle activation patterns during walking were recorded using surface electromyography (EMG) (Delsys, Boston MA; Bagnoli-8, DE 2.1 electrodes) from the left *vastus lateralis* (VL), *biceps femoris* (BF), medial *gastrocnemius* (GA), and *tibialis anterior* (TA) [16]. The EMG data were sampled at 1080 Hz, bandpass filtered (passband 20–300 Hz in software; built-in 20–450 Hz hardware filter), notch-filtered at 60 Hz using zero-lag Butterworth filters, and demeaned using MATLAB 7.04 (Mathworks, Natick MA).

Data from some trials were discarded from VL (15 of 349 collected), BF (17/349), GS (8/349), and TA (7/349) muscles due to signal quality issues. Also, noisy sections of the signals due to sweating, etc., were identified visually and were not used in subsequent analyses.

### Multiscale Entropy Calculations for Surface EMG

The complexity of each EMG signal was quantified using the multiscale entropy (MSE) method described in detail elsewhere [17]. Briefly, MSE quantifies the degree of irregularity of time series data over multiple time scales. Time series data that are more irregular or entropic over a broad range of time scales are considered more complex than those that show irregular behavior at only a single time scale. Single-scale methods, such as approximate entropy and sample entropy (SampEn), yield higher entropy values for uncorrelated random signals than for signals with long-range correlations, especially with signals of high sampling frequency (i.e., small time scales). However, the reverse occurs at larger time scales, as long range patterns do not exist in uncorrelated noise and yield low entropy values, yet signals with long-range correlations still contain dynamics at these larger timescales [17]. EMG signals exhibit fairly uniform power spectrum throughout the passband. This power spectrum is similar to that of filtered uncorrelated white noise. Both signals display decreasing sample entropy with increasing timescales. Despite their “noise”-like spectral properties, EMG signals contain important information about muscle activation patterns [18].

Physiologically, surface EMG during gait is comprised of the filtered sum of action potentials from the muscle fibers, describing an overall “ensemble” effect of the activation of different motor units. Each motor unit contributes to the overall signal, and therefore its activation can add to the overall complexity of the surface EMG signal. During muscle activation, motor units cycle on and off, and their firing rates can change. We expect that physiological changes associated with aging, such as loss of motor units, to reduce the number of elements that contribute to the overall EMG signal, and thus produce a less complex output. This result is predicted by the multisystem dysregulation hypothesis [5]. However, both motor unit firings and firing patterns can become more variable, which could therefore cause the overall signal to be less predictable and therefore exhibit higher entropy. These different physiological mechanisms may explain the discrepancies observed between different previous studies.

In addition, multiscale relationships between EMG signal dynamics and time scales on the results has not been adequately considered in many studies of aging motor function, yielding seemingly conflicting results as they tend to compare different timescales [10–14]. In contrast, the MSE method considers SampEn over a range of timescales, and provides a more complete picture of the signal properties, and thus could clarify this issue.

The MSE analysis consists of three steps: 1) coarse-graining the original time series to derive multiple signals, each capturing the system dynamics at different scales; 2) calculating a measure of entropy suitable for finite time series, SampEn in this case, for each coarse-grained time series; and 3) integrating the entropy values over a pre-defined range of scales to obtain an index of complexity (C_I_). For a time series [*x*_1_, *…*, *x*_*N*_], each element *j* of the coarse-grained time series *y* for scale *n* was calculated according to the Eq 1.

$$y_{{}^{j}}{}^{\left(n\right)}=\frac{1}{n}\overset{jn}{\underset{i=(j-1)n+1}{\sum }}x_{i}$$ (1)

**Fig 1:**
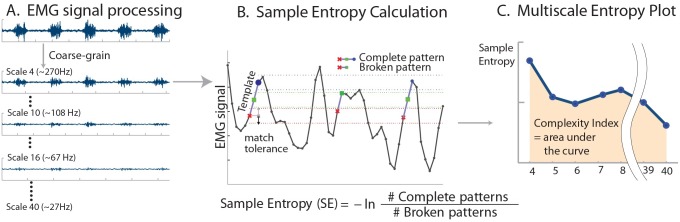
Schematic of Multiscale Entropy Calculation. (A) After the original EMG signal is bandpass filtered at 20–300 Hz, it is coarse grained to extract dynamics at different time scales. (B) Sample entropy (SampEn) is calculated from each coarse grained signal. For each pattern of *m* points in the signal (template example: ×-■), places in other parts of the signal where the template is seen are identified within tolerance *r*. SampEn is calculated as the negative natural log (-ln) of the conditional probability that the pattern of *m*+1 points (×-■-○) will match if that the pattern of *m* points (×-■) did match. In other words, after the signal matched the first two parts of the pattern ×-■, this is the probability that pattern match will complete, ×-■-○. The number of ×-■ matches are compared to the number of complete pattern (×-■-○) matches. Higher SampEn indicates that the signal is less predictable, and thus more irregular. (C) Multiscale view of the signal is derived by examining sample entropy of the EMG at each of the coarse-grained time scales. Complexity index C_I_ is defined as the area under the curve. Higher C_I_ indicates that the signal has unpredictable dynamics over a wide range of time scales, and thus more complex.

MSE, as noted, uses sample entropy to quantify the degree of irregularity of a time series. Sample entropy is a conditional probability measure that quantifies the likelihood that if a vector with *m* data points matches a template with the same length, then the vector and template will still match when their length increases from *m* to *m*+1 data points, within a given tolerance *r*. SampEn can theoretically vary from 0 to infinity, but generally ranges between 0 and 3 in the literature [17]. Plotting sample entropy for each coarse-grained time series as a function of time scale yields the MSE curve (Fig 1). The complexity index (C_I_) is the area under the MSE curve [17] that indicates the amount of information, or entropy, in a signal over a certain range of time scales. Consistently high entropy values over a wide range of time scales, and thus high C_I_, indicate high complexity, and vice versa [17]. The length of the original time series determines the largest scale that can be analyzed [17]. Here, C_I_ was calculated using the trapezoid rule.

This use of multiple entropy method uses the same amount of tolerance *r* for all of the timescales. For signals that do not exhibit dynamics at larger timescales, coarse graining will produce signals with low amplitudes relative to *r*, and that are thus more “regular” or of less entropy. Depending on the timescales in which complex dynamics can be observed, two different signals can produce the same overall complexity index. Thus, scale-by-scale comparisons, as performed here, are also useful.

### Sample Entropy Parameter Choices

Parameter choices in entropy calculations can affect results, particularly in short data sets [19]. We used scales *n* = 4–40, template length *m* = 2 and tolerance *r* = 0.15 of the standard deviation of the processed EMG signal, using previous recommendations [17, 19–21]. Scales *n* = 4 through 40, equivalent to 1080Hz/4 = 270Hz through 1080Hz/40 = 27 Hz, were chosen to match the 20–300 Hz passband, although larger scales could be analyzed given the length of the time series (324,000), EMG dynamics at lower or higher frequencies did not exist in the signal after the bandpass filtering. Due to the high-pass hardware filtering at 20 Hz, analyses were not extended to larger timescales beyond *n* = 40 or 50 ms (or lower than 20 Hz) as larger time scale dynamics were removed by filtering. Likewise, dynamics at scale 1 (1080 Hz), scale 2 (540 Hz), and scale 3 (360 Hz) were not used as they would not contain information about the EMG signal after it was filtered [7], and to avoid any potential issues with oversampling. Template length *m* = 2 was chosen for computational expediency, in line with previous recommendations [18]. Tolerance *r* = 0.15 SD was chosen a priori based on literature.

Although entropy measures are known to be sensitive to the choice of *r* for short data sets [19], we used long data sets to minimize the effect of this particular parameter choice. We also repeated the analysis for *r* = 0.01, 0.05, 0.25, and 0.35 SD, as well as *m* = 3 (and the same range of *r*). For computational expediency, these analyses were completed for scales *n* = 4, 8, 12, 16, 20, 24, 28, 32, 36, and 40. Trapezoid rule was used to calculate C_I_ to make the comparisons similar to studying scales *n* = 4–40. Signal processing was performed using MATLAB 2016a (Mathworks, Natick MA) on Amazon EC2 cloud cluster.

### Phasic Bursts and Non-stationarity in EMG

Because EMG signals during walking have strong phasic bursts during each stride, the EMG signal is locally non-stationary. This posed unique challenges in adapting the multiscale entropy method to the gait EMG signal. This non-stationarity can pose a significant problem in estimating entropy using the template-matching approach explained above. As the signal mean or the amplitude deviate from another part of the signal, the signal will no longer match the template. If only a few template matches occur, our estimate of the conditional probability of template matches would be inaccurate. For an accurate estimate of sample entropy, enough of the non-matches and matches need to occur. We addressed the non-stationarity of the signal mean with a high-pass filter in the post-processing and through using large amount of data.

Detrending is a common method to remove the non-stationarity of the signal mean, particularly in short data sets [17]. Detrending was unavoidable in our study due to the built-in high-pass hardware filter and limited our ability to study longer timescales beyond 20 Hz or 50 ms. This high pass process minimized the fluctuation of the signal mean from one burst to another, and made the signal mean stationary across multiple strides. Another approach to create stationary amplitudes that has been used in the literature is to control the experimental protocol to very simple movements [18]. As this was not appropriate for studying gait, we instead collected a large quantity of continuous data (5 minutes @ 1080 Hz = 324,000 data points in a time series). After coarse graining, the time series lengths were between 8100 (= 324,000/40 for scale 40) to 81,000 (= 324,000/4 for scale 4), much longer than the recommended minimum of 150–200. Using long time series data, we ensured that each EMG signal contained enough bursts associated with enough strides, so that each signal was stationary in the large scale, and thus enough template matches would occur to accurately estimate sample entropy.

#### EMG Duty Factor and the Non-stationarity of Amplitude

Aside from non-stationarity of the signal mean, the non-stationarity of the amplitude (or variance) is another issue. Due to the phasic bursts, signal amplitudes are not stationary in EMG signals during gait due to gaps of minimal muscle activity between bursts between gait cycles. Likewise, any large spikes in the data posed the same problem, as the overall signal amplitude, as measured using the standard deviation is inflated by these spikes. Both of these issues make the matching tolerance large particularly in the low-amplitude quiescent portions of the EMG signal. Because the local signal amplitude is quite small compared to the large tolerance, many more template matches can occur and thus the signal will exhibit lower entropy. As this is the nature of the EMG signals during gait, this issue is unavoidable.

Therefore, in preliminary work, we quantified the effect of burst duration vs. quiescence duration on the complexity index (C_I_) in a pilot simulation to better understand the potential impact of this effect on C_I_ calculations. We called the proportion of the EMG “on” during the gait cycle “EMG duty factor”. Since aging is associated with longer burst durations, this could potentially confound our interpretation of the final results. To assess the possible effects of EMG duty factor on the complexity index, simulated EMG signals were created as bandpass filtered white noise (20–300 Hz, same settings used for recorded signals). Of note, although filtered white noise does not model all aspects of EMG signals during gait, it is used here because (1) it contained similar power spectra and multiscale entropy profiles within the passband, and (2) it allowed us to test the effect of EMG duty factor with other things being constant.

**Fig 2:**
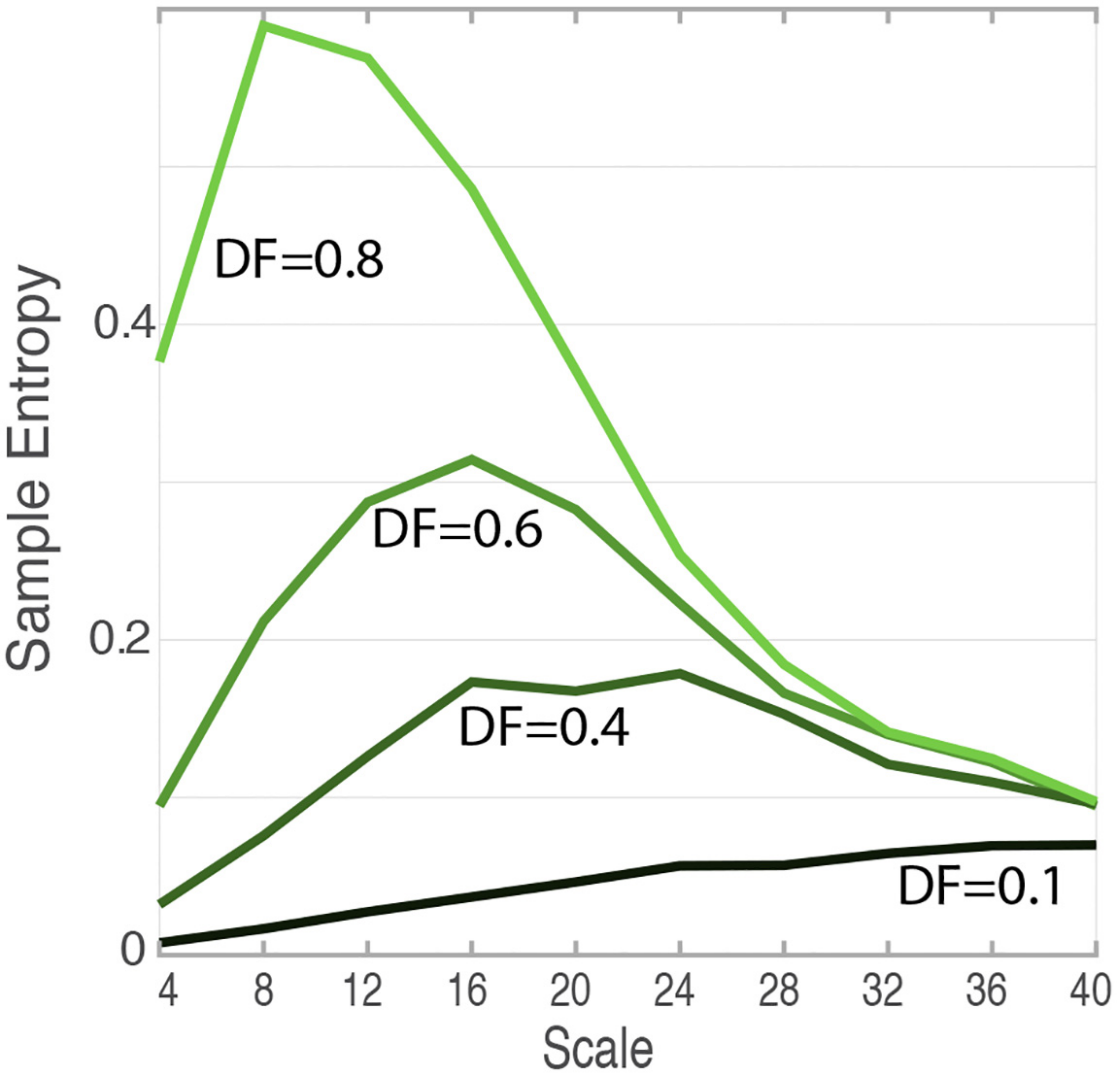
Effect of EMG duty factor on the Multiscale Entropy of Simulated EMG. Increasing EMG duty factor of EMG signals increased the SampEn of the EMG signal at the smaller timescales. This is because the signal is smoother on average in the short timescales, since the quiescent portions become very predictable as templates will match very often. In real EMG signals, EMG duty factor could confound the observed complexity differences between two signals.

Simulated EMG time series of same lengths (324,000) were created. Within each “gait cycle” of 1.4 seconds, regular gaps were inserted to create signals of 10, 40, 60, and 80% EMG duty factor, where the muscle was “active” for 10–80% of the gait cycle, throughout the simulated 5 minutes of walking. Simulation results indicated that decreasing EMG duty factor (shorter EMG bursts within a gait cycle) led to lowering of sample entropy values during short timescales (Fig 2). Therefore, the EMG duty factors in the recorded EMG signals were calculated as a possible confounding variable.

This positive correlation of C_I_ with EMG duty factor was as expected based on the method of multiscale entropy. With decreasing burst durations and EMG duty factor, there are more “off,” “flat,” “regular,” and thus predictable parts to the signal. More numbers of pattern matches and completions will occur during these quiet periods, since the signal looks very predictable during these “off” parts. With more pattern completions, the sample entropy of the signal would be less, thus would lead to overall lower C_I_.

### EMG Duty Factor Calculation

**Fig 3:**
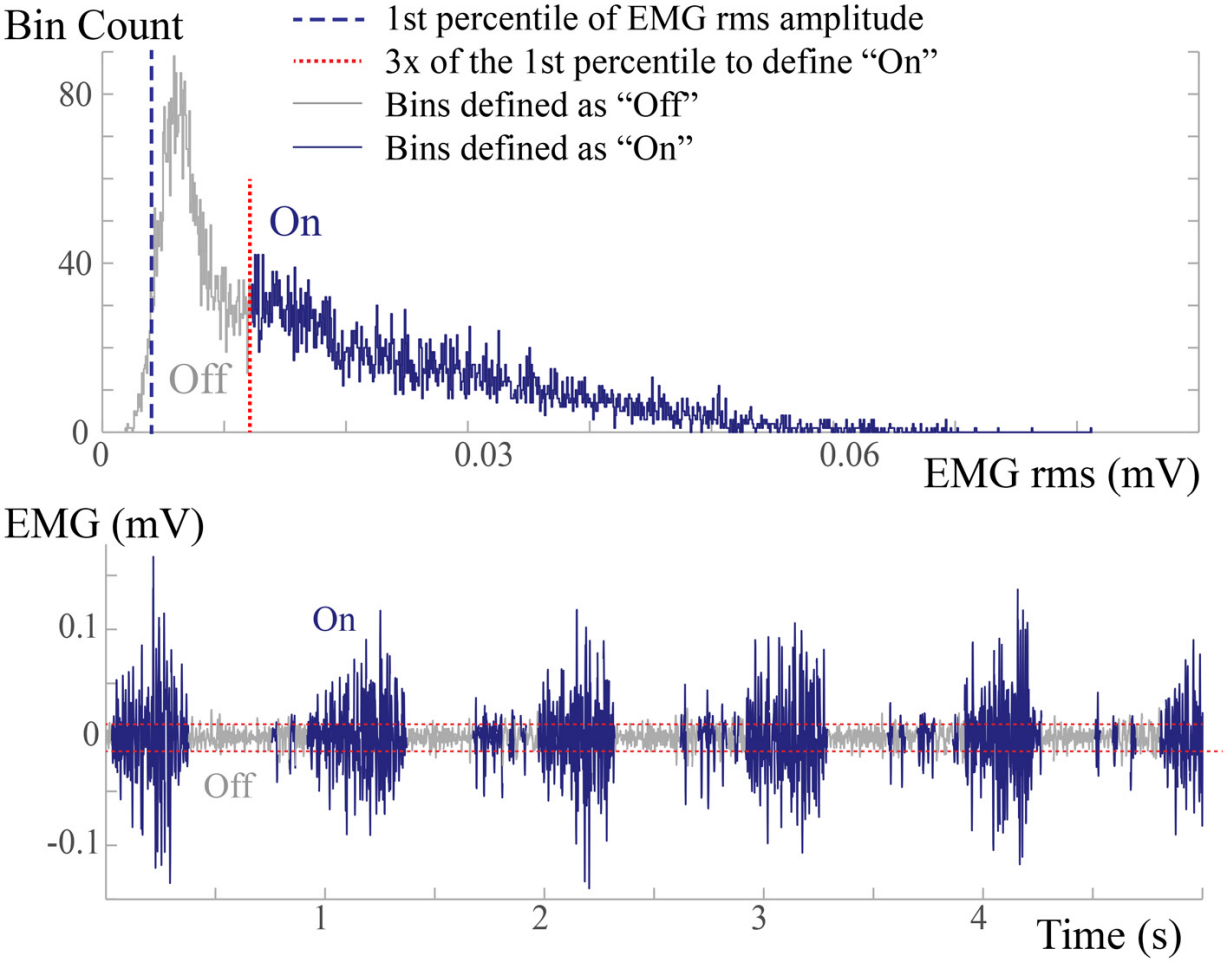
EMG duty factor Calculation. (A) RMS amplitude histogram. The EMG signal was divided into 25-ms portions and the root-mean-square (RMS) amplitude was calculated. Amplitude histogram is shown in (A). The 1^st^ percentile was used to assume that this EMG activity is when the muscle is quiescent or “Off.” “On” was defined as 3× the RMS amplitude. (B) Sample EMG signal denoted with On/Off times. “On” portion is marked in dark blue; “Off” portion is marked in grey. This method identifies phasic bursts as “On” relative to the quiescent “Off” periods.

To calculate the EMG duty factor in the recorded EMG signals, the signal was divided into 25-ms epochs, and then RMS amplitude of each epoch was calculated. We made the assumption that the lowest 1% of RMS amplitudes would definitely occur when the muscle is “off.” The initial cutoff amplitude that divides the epochs with bottom 1% of the amplitudes from those in the top 99% was identified. Then, the muscle was defined to be “on” if the RMS amplitude of the epoch greater than three times of the initial cutoff (Fig 3).

### Statistics

First, to test whether older adults exhibited lower complexity and whether this effect was attenuated by slower walking speed, we compared the complexity index C_I_ between age groups and across walking speeds using a general linear model analysis of variance (ANOVA) for each of the four muscles (proc glm, subjects nested within age groups as a random factor). Analyses were repeated across *m* = 2–3 and *r* = 0.01–0.35 SD. EMG duty factors were also compared likewise. Second, C_I_ was compared between age groups and across walking speeds with EMG duty factor as a continuous covariate, in an analysis of covariance (ANCOVA; using proc glm). Third, for a comprehensive look at the multiscale nature of EMG signals, we compared sample entropy between the age groups and speed at each timescale with α = 0.001 (≈0.05 / 37 scales) to account for multiple comparisons. SAS 9.3 was used (SAS Institute, Cary NC).

## Results

### Unadjusted Complexity Indices

**Fig 4:**
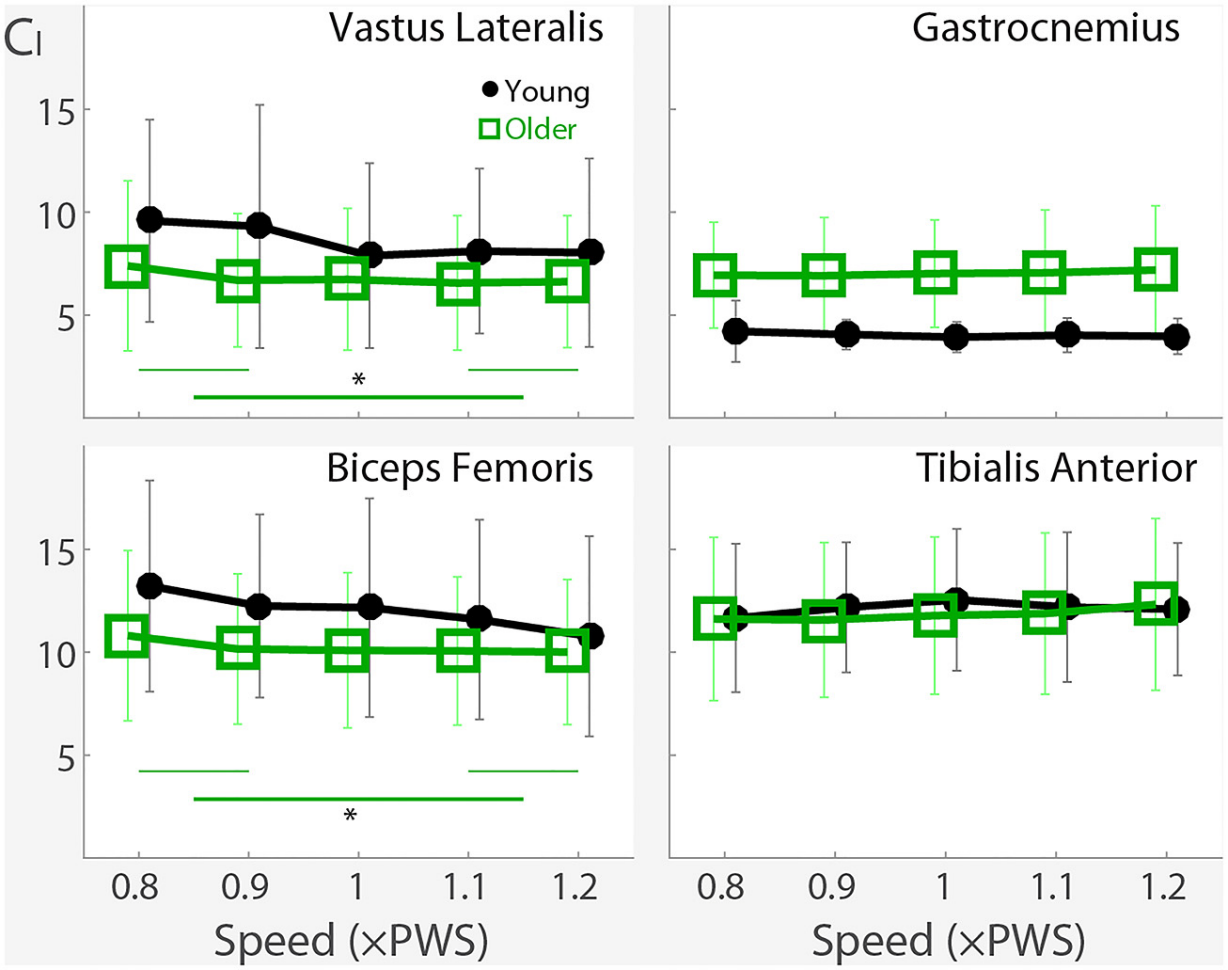
Age and Walking Speed Effects on the Complexity Index. The (unadjusted) complexity index C_I_ are shown for *vastus lateralis*, *biceps femoris*, *gastrocnemius*, and *tibialis anterior* muscles as a function of the age group and walking speed. Error bars indicate standard deviation. C_I_ decreased slightly with increasing walking speed in the proximal muscles. P-values are shown in the plot. Significant (p<0.05) pairwise Tukey-Kramer post-hoc tests are indicated with an asterisk (*). In *vastus lateralis* and *biceps femoris*, the two slow speeds were significantly different from the two fast speeds. After covariate adjustment with EMG duty factor, age-group differences were no longer present in *biceps femoris* (p = 0.06), but became noticeable in *tibialis anterior* (p<0.0001).

The complexity indices C_I_ in *vastus lateralis* (VL) (p < 0.0001) and *biceps femoris* (BF) (p<0.0001) were lower in older adults (Fig 4). However, in *gastrocnemius* (GA), older adults exhibited greater complexity C_I_ (p<0.0001). In *tibialis anterior* (TA), the effect of age was not significant (p = 0.14). A small decrease in the C_I_ was observed with increasing walking speed in VL (p<0.0001) and BF (p = 0.0003; Fig 4), where the two slow speeds were significantly different from the two fast speeds (p<0.05), per pairwise Tukey-Kramer post-hoc tests. Effects of walking speed were not observed in GA or TA.

### EMG Duty Factors

**Fig 5:**
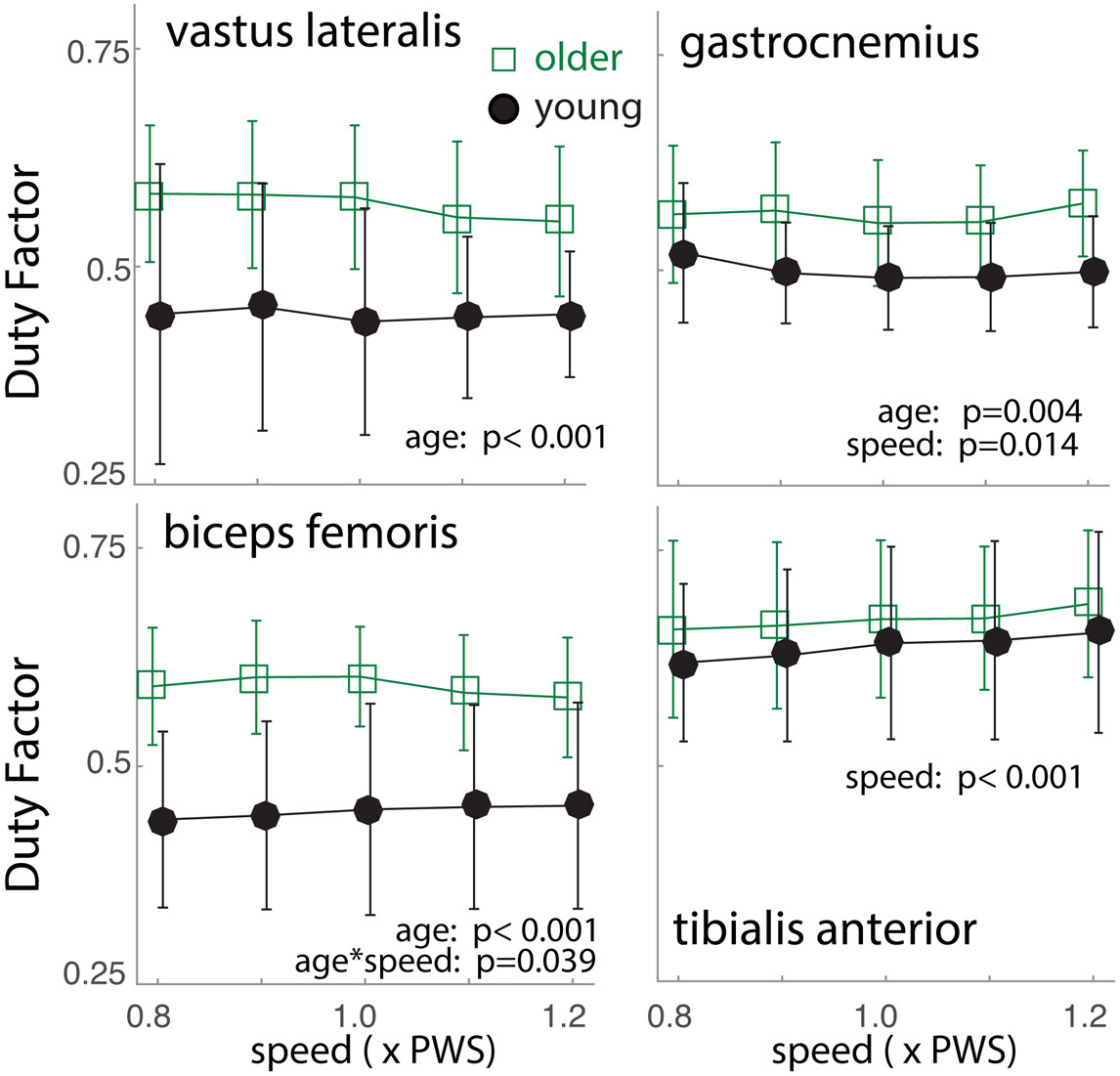
Age and Walking Speed Effects on EMG duty factor. The fraction of the time that *vastus lateralis*, *biceps femoris*, *gastrocnemius*, and *tibialis anterior* muscles as “on” within a gait cycle is plotted as a function of the age group and walking speed. Error bars indicate standard deviation. Older adults exhibited higher EMG duty factor than young adults except in *tibialis anterior*. Statistically significant P-values are shown in the plot.

EMG duty factors were higher in older adults in VL, BF (p<0.001) and GA (p = 0.004), but not in TA (p = 0.33). Group differences were more noticeable at slower walking speeds in BF (interaction p = 0.039). A slight quadratic relationship was observed in GA with walking speed, where the EMG duty factor was the lowest at the preferred speed (p = 0.014). TA EMG duty factor increased slightly with speed (p<0.001; Fig 5).

EMG duty factors were negatively correlated to C_I_ in VL (Pearson r = -0.407, p<0.0001) and BF (r = -.272, p<0.0001), and positively correlated in GA (r = 0.184, p = 0.0007) and TA (r = 0.34, p<0.0001). Correlations strengths were “very weak” (|r|<0.2), “weak” (0.2≤|r|<0.4) to “medium” (0.4≤|r|<0.6) [22]. These were in contrast to the simulated results, where EMG duty factor and C_I_ were positively correlated.

### Covariate Adjusted Complexity Indices

Since EMG duty factors were associated with age, speed, and C_I_, C_I_ was compared after covariate adjustment. After adjusting for EMG duty factor, the C_I_ for VL were higher in young adults as before (p = 0.0005), but no longer statistically significant in BF (p = 0.06), although the trends were similar. C_I_ in GA was higher in older adults as before (p<0.0001). After adjustment, C_I_ in TA was higher in young adults (p<0.0001). The effect of walking speed remained similar: C_I_ decreased with speed in VL (p<0.0001) and BF (p = 0.0004), but not in GA (p = 0.89) or TA (p = 0.95). The results indicate that although EMG duty factor is associated with C_I_, age, and speed, it only affects the results in BF and TA.

### Parameter Choices

These unadjusted results were generally consistent across multiple *m* and *r* values, with the exception of *m* = 2 and *r* = 0.05 SD for the GA, where the young adults exhibited higher C_I_ (Table 2). Smaller *r* values were more sensitive to age and speed differences, whereas these differences were not observable with larger *r* values, particularly in BF and TA. Covariate adjusted results were in general similar to the unadjusted results, except at *m* = 2 and *r* = 0.15 SD as discussed above.

**Table 2:** Complexity Index Comparisons across *m* = 2–3 and *r* = 0.01–0.35 SD with and without Covariate Adjustment.

*m* = 2				
*r (×SD)*	**Vastus lateralis**	**Biceps femoris**	**Gastrocnemius**	**Tibialis anterior**
**0.01**	Young>Older (Y>O), p<0.0001	Y>O, p<0.0001	O>Y, p<0.0001	Y>O, p<0.0001
	Slow>Fast (S>F), p<0.0001	S>F, p<0.0001	S<>F, p = 0.23	S<>F, p = 0.17
	After covariate adjustment			
	Y>O, p<0.0001	Y>O, p = 0.002	O>Y, p<0.0001	Y>O, p<0.0001
	S>F, p<0.0001	S<>F, p = 0.24	S<>F, p = 0.9	S<>F, p = 0.09
**0.05**	Y>O, p<0.0001	Y>O, p<0.0001	Y>O, p<0.0001**	Y>O, p<0.0001
	S>F, p<0.0001	S>F, p<0.0001	S<>F, p = 0.41	S<>F, p = 0.40
		Ix p = 0.04*		
	After covariate adjustment			
	Y>O, p<0.0001	Y>O, p<0.0001	Y>O, p<0.0001**	Y>O, p<0.0001
	S>F, p<0.0001	S>F, p<0.0001	S<>F, p = 0.43	S<>F, p = 0.37
		Ix p = 0.02		
**0.15**	Y>O, p<0.0001	Y>O, p<0.0001	O>Y p<0.0001	Y<>O p = 0.14
*a priori* recommendation	S>F, p<0.0001	S>F, p = 0.0003	S<>F, p = 0.91	S<>F, p = 0.22
(see text)	After covariate adjustment			
	Y>O, p = 0.0005	Y>O, p = 0.0624	O>Y, p<0.0001	Y>O, p<0.0001
	S>F, p<0.0001	S>F, p = 0.0004	S<>F, p = 0.89	S<>F, p = 0.95
**0.25**	Y>O, P<0.0001	Y>O, p<0.0001	O>Y, p <0.0001	O<>Y, p = 0.93
	S>F, p = 0.0003	S>F, p = 0.02	S<>F, p = 0.93	S<>F, p = 0.43
	After covariate adjustment			
	Y>O, P = 0.0001	Y>O, p<0.0001	O>Y, p < 0.0001	O<>Y, p = 0.86
	S>F p<0.0001	S>F, p = 0.02	S<>F, p = 0.93	S<>F, p = 0.45
**0.35**	Y>O, p<0.0001	Y>O, p = 0.0007	O>Y, p<0.0001	O<>Y, p = 0.47
	S>F, p = 0.007	S<>F = 0.27	S<>F, p = .88	S<>F, p = 0.54
	After covariate adjustment			
	Y>O p<0.0001	Y>O, p = 0.0007	O>Y, p<0.0001	O<>Y, p = 0.66
	S>F, p = 0.002	S<>F = 0.29	S<>F, p = 0.90	S<>F, p = 0.55
*m* = 3				
*r*	**Vastus lateralis**	**Biceps femoris**	**Gastrocnemius**	**Tibialis anterior**
**0.01**	Y>O, p<0.0001	Y>O, p = 0.0001	O>Y, p<0.0001	Y>O, p<0.0001
	S>F, p<0.0001	S>F, p = 0.011	S<>F, p = 0.24	F>S, p = 0.04***
	After covariate adjustment			
	Y>O, p<0.0001	Y>O, p<0.0001	O>Y, p<0.0001	Y>O, p<0.0001
	S>F, p<0.0001	S>F, p<0.0001	S<>F, p = 0.4244	F<>S, p = 0.18
**0.05**	Y>O, p<0.0001	Y>O, p<0.0001	O>Y, p<0.0001	Y>O, p<0.0001
	S>F, p<0.0001	S>F, p = 0.0005	S<>F, p = 0.55	F>S, p = 0.04***
	After covariate adjustment			
	Y>O, p<0.0001	Y>O, p<0.0001	O>Y, p<0.0001	Y>O, p<0.0001
	S>F, p<0.0001	S>F, p<0.0001	S<>F, p = 0.551	F<>S, p = 0.10
**0.15**	Y>O, p<0.0001	Y>O, p<0.0001	O>Y, p<0.0001	O<>Y, p = 0.13
	S>F, p<0.0001	S>F, p = 0.005	S<>F p = 0.83	S<>F p = 0.10
	After covariate adjustment			
	Y>O, p = 0.0023	Y>O, p<0.0001	O>Y, p<0.0001	Y>O, p = 0.04
	S>F, p<0.0001	S>F, p<0.0001	S<>F, p = 0.84	F<>S, p = 0.19
**0.25**	Y>O, p<0.0001	Y>O, p<0.0001	O>Y, p<0.0001	O<>Y, p = 0.83
	S>F, p = 0.0005	S<>F, p = 0.12	S<>F p = 0.89	S<>F, p = 0.20
	After covariate adjustment			
	Y>O, p = 0.004	Y>O, p<0.0001	O>Y, p<0.0001	O<>Y, p = 0.68
	S>F, p<0.0001	S>F, p = 0.0072	S<>F, p = 0.87	S<>F, p = 0.35
**0.35**	Y>O, p<0.0001	Y>O, p<0.0005	O>Y, p<0.0001	O<>Y, p = 0.36
	S>F, p = 0.007	S<>F, p = 0.64	S<>F, p = 0.92	S<>F, p = 0.33
	After covariate adjustment			
	Y>O, p = 0.0007	Y>O, p = <0.0001	O>Y, p<0.0001	O<>Y, p = 0.74
	S>F, p = 0.0004	S<>F, p = 0.1607	S<>F, p = 0.87	S<>F, p = 0.48
**Summary**	Young>Older and Slow>Fast for all parameters	Young>Older and Slow>Fast for r<0.25SD	Older>Young for all, except m = 2 and r = 0.05SD	Young>Older for r<0.15SD
			No speed effect for all parameters	No speed effect for all parameters, except m = 3, r≤0.05

*Ix: Age-speed interaction was statistically present, where the speed effect was stronger in young adults.

** The only exception to the trend in the *gastrocnemius*

*** Although statistically significant at α = 0.05, these findings are marginal considering the large number of comparisons made. A Bonferroni adjustment would negate these effects.

Y>O: C_I_ in young adults higher than in older adults

O<Y: C_I_ in older adults higher than in young adults

S>F: C_I_ in slow speeds higher than in fast speeds

F>S: C_I_ in fast speeds higher than in slow speeds

Y<>O, S<>F: age effect or speed effect not statistically significant at α = 0.05

Covariate adjustment did not affect most parameter sets, except for *biceps femoris* and *tibialis anterior* at *m* = 2, *r* = 0.15SD (see text).

### Entropy and Timescales

**Fig 6:**
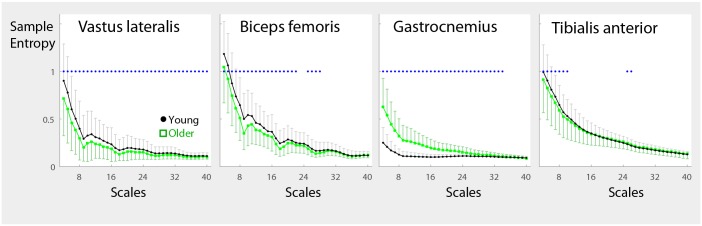
Age and Timescale Effects on the Sample Entropy. Sample entropy of the EMG signals for *vastus lateralis*, *biceps femoris*, *gastrocnemius*, and *tibialis anterior* were calculated for timescales 4–40 (27–270 Hz) after successive coarse-graining. The timescales and corresponding frequencies are shown on the abscissa of the plots. Error bars indicate standard deviations. SampEn values in general become smaller at larger timescales. Age-related differences are more noticeable at small timescales. Older adults exhibited lower SampEn values in the proximal muscles, and higher values in the *gastrocnemius*. Significant age-related differences (main effect of age) at α = 0.001 (≈ 0.05/37 scales tested) are shown with the dot (∙) above each timescale. Plotted mean and standard deviation values were pooled across walking speeds.

**Fig 7:**
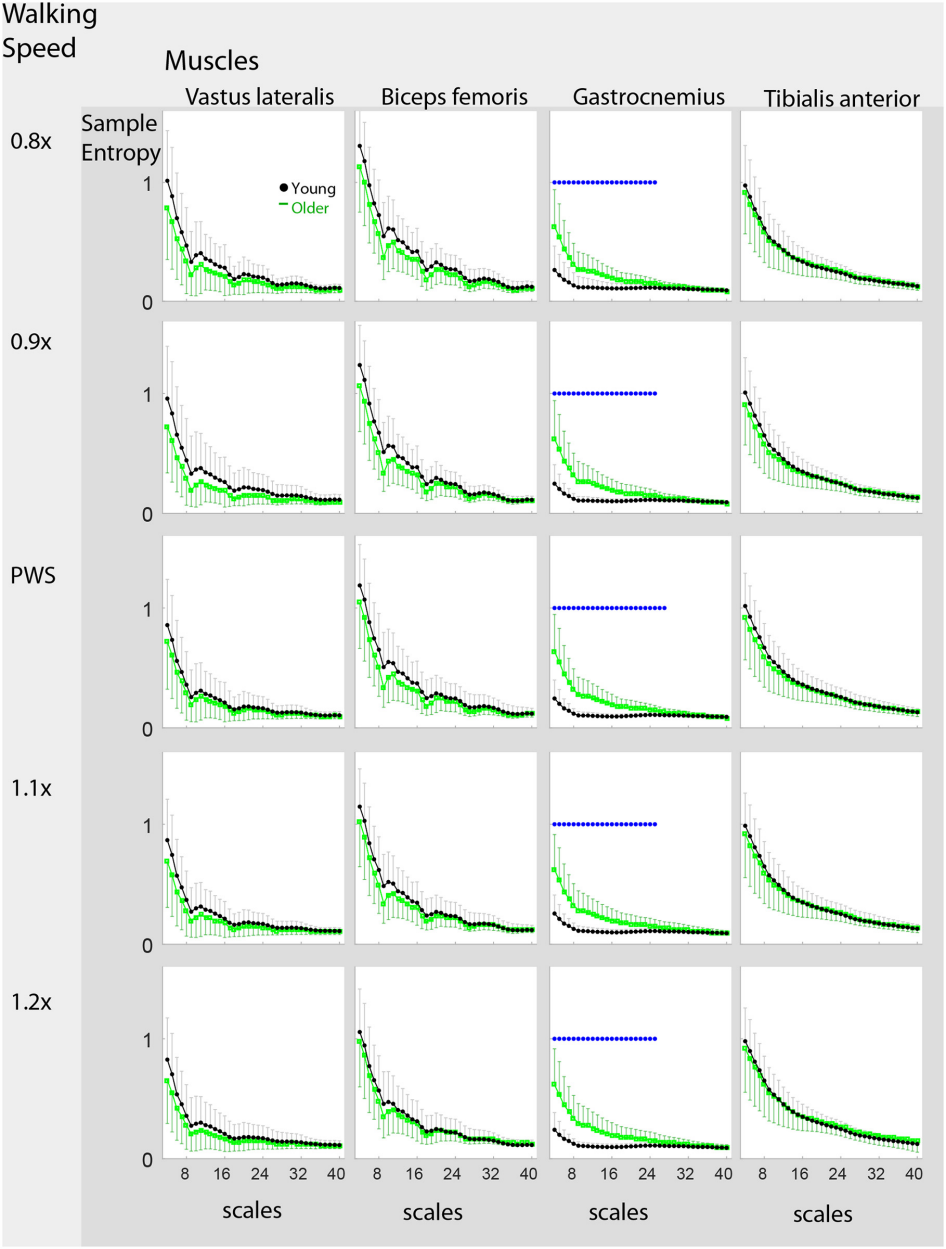
Age and Timescale Effects on Sample Entropy stratified by Speed. Sample entropy values are shown stratified by walking speed. Although the main effect of walking speed was present as shown in Fig 4, the trends between walking speeds are similar. The asterisk (*) denotes significant age-related differences as determined using Welch’s t-test (unequal variances, 2-tail). Compared to Fig 6, the age differences are not statistically significant except with *gastrocnemius* due to the less powerful t-test and the smaller sample size.

Young adults exhibited greater sample entropy (*m* = 2, *r* = 0.15 SD) at most timescales in VL and BF, and at small timescales in TA (p<0.001). The opposite was observed in the GA. Age-related differences were more noticeable at small timescales (Figs 6 and 7). Sample entropy overall decreased with increasing timescales. However, the GA in young adults exhibited a nearly flat profile. The effect of walking speed was similar to that in Table 2. Age-group differences were similar across walking speeds (Fig 7).

## Discussion

Our goals were to determine whether older adults exhibit lower overall complexity of muscle activation patterns by studying the dynamics of EMG patterns over multiple time scales, and whether slower walking would attenuate this effect. We observed a lower complexity index of EMG activations during gait in older adults, but only in *vastus lateralis*, *biceps femoris* and *tibialis anterior* with smaller *r*. The opposite was observed in the *gastrocnemius*, and this effect was more pronounced at the shorter timescales and smaller *r*. These results were generally robust across different sample entropy parameters, and after adjusting for EMG duty factor. Slower walking speed produced higher complexity in the proximal muscles, but not in the distal muscles. We conclude that the decrease in complexity in EMG signals with aging may be limited to proximal leg muscles, particularly the *quadriceps*, and that slower walking attenuates this decrease in complexity in the timescales (27–270 Hz) studied. This finding was observed despite longer EMG burst durations within the gait cycle as observed in older adults, which would have increased rather than decreased C_I_. Therefore, contrary to the multisystem dysregulation hypothesis of aging, greater complexity may also indeed be a sign of deterioration, at least within our analysis range of 27-270Hz in some systems. This may not apply to behaviors occurring in other timescales.

Older adults exhibited lower complexity particularly in short timescales as well as longer burst durations. Our pilot simulation indicated that longer bursts would produce higher complexity values, but this relationship was opposite in the proximal muscles. Therefore, we conclude that in the quadriceps, the decreases in complexity are not due to simple changes in burst durations, but despite them. *Biceps femoris* also exhibited lower complexity in older adults, but this may be due to changes in activation burst patterns rather than changes in the EMG signal complexity, since changes in EMG duty factor could explain the age group differences. However, in the *gastrocnemius* the longer burst may explain the increased complexity, and in *tibialis anterior* the age-related decrease in complexity may be masked by the longer burst durations.

### Aging and Complexity

The lower complexity in older adults observed in the proximal muscles was predicted by the multisystem dysregulation hypothesis. In the motor system, this result may be explained by sarcopenia and the associated loss of motor units [23, 24]. As the surface EMG signal is created from a smaller number of motor units, and therefore the resulting signal would be less complex [23]. Age-related differences in SampEn values were more notable in the small timescales and SampEn values of older adults became lower than that of young adults at very large timescales, although the differences were minimal (Fig 3).

However, sarcopenia by itself does not explain this phenomenon, as the opposite was found in the *gastrocnemius*. Sarcopenic deterioration with aging is well known in plantarflexor muscles, as well as proximal muscles [24]. Also, the fiber composition is likewise similar in the *vastus lateralis* of young and older adults [25]. Therefore, sarcopenia may explain the results of proximal muscles, but not others.

One possible explanation for this difference between the muscles is the substantial change in their activation pattern with aging. The activation of the *gastrocnemius* may change to somehow compensate for the loss of complexity in the proximal muscles. During gait, older adults exhibit increased hip moments and decreased ankle moments [26] and power [27], and activate proximal muscles more [4, 28]. Yet distal muscles activate more during balance challenged walking [29] in older adults, and this may be the case in our study, where the treadmill walking task without handrails was somewhat novel to the older adults. We observed that burst durations were longer in older adults, and this was associated with increased C_I_.

Another possible explanation for our result is the increase in neuromuscular noise with aging. As older adults activate proximal leg muscles more [4, 28], there may be less motor drive to distal muscles. With increased neuromuscular noise and less drive ‘signal’ in the *gastrocnemius*, the noise portion may be dominating the entropy characteristics of the EMG output. Sample entropy of white noise are higher at smaller timescales [17], similar to the EMG signals we observed in the *gastrocnemius* (Fig 3). This may explain the unique results in the *gastrocnemius* of older adults. Also, the positive correlation between EMG duty factor and C_I_ were similar to that of white noise. Therefore, signal complexity profile may represent neuromuscular noise rather than the changes in drive signal itself, as multiscale entropy was found to increase only minimally with increasing isometric contraction intensity [18].

A third possibility is the longer EMG burst durations during walking. Although our analysis does not consider longer timescales that would capture the stride-to-stride fluctuations of muscle burst times or lengths, we did separately calculate the burst durations by way of calculating the EMG duty factor. As our pilot simulation indicated that higher EMG duty factor would produce higher complexity, and this effect was seen in the *gastrocnemius*. The higher EMG duty factor in the *gastrocnemius* may partially explain the increased in complexity in older adults. This result is congruent with the reported increased co-contraction [4] and burst duration.

In consideration of these three possible explanations, we speculate that the muscle fiber activation in the *gastrocnemius* becomes more like that of white noise. Given the known progression of sarcopenia, proximal muscles may also show this tendency as aging continues.

### Gait Speed and Complexity

Slow gait in older adults may reflect adaptations to manage the walking task. We examined whether slower walking speed would minimize age-related differences in complexity. Although the age-related differences were not different across walking speeds, faster walking speed led to lowering of the complexity of EMG signals in the proximal muscles. This result supports our hypothesis that older adults may be benefitting by walking slower, and that slower walking may be an adaptive behavior. Certainly, there are physical limitations associated with aging and frailty that can limit walking speed. Nonetheless, we speculate that walking slower may allow the nervous system to function with higher complexity, which may allow for better function. Walking speed is known to produce changes in stepping control and timing, which likely occur in larger timescales (~0.1–1 Hz) not reflected in our analysis, limited to 27–270 Hz. EMG and other gait dynamics at these timescales may need to be examined to better address this question of adaption.

### EMG Signals and Complexity

Ours may be the first work to examine the entropy of muscle activation patterns during gait in a comprehensive multiscale manner. To accomplish this, we measured EMG over 5 minutes of walking, which provided sufficient data to study dynamics that are present over the frequency range of EMG. Previous studies have used a wide range of methods that may have led to the apparent conflicts in the results [10–14], or are limited to isometric contractions [18, 30]. Methodologically, we recommend reporting entropy values over the range of timescales. It is particularly important to study only the timescales that fall within of the passband of the filtering methods used, consider the effects of low frequency non-stationarity on entropy estimates, and confounding effects of burst durations.

EMG signals during gait exhibited larger sample entropy at small timescales, and lower entropy at larger timescales. This behavior was similar to that of bandpass filtered uncorrelated white noise. Both EMG and filtered noise have similar power spectrum over the frequencies considered, which may explain this similarity. Practically, it may not be necessary to consider the larger timescales considered in our study. Timescales from 4 to 30 ms may be adequate for future studies, but studying larger scales may be useful for understanding stride-to-stride fluctuations.

### Limitations and Future Work

Our work is descriptive of neuromuscular behavior, and does not explain the sources of the complexity of the signal or their mechanistic changes due to aging. Our work is also limited in studying the passband of the surface EMG signal. Only one decade of timescales (27–270 Hz) was considered, due to the nature of the surface EMG signals. Although the timescales and the dynamics studied are not strictly defined by linear behavior of the signal at each frequency, analyses of timescales beyond the passband will likely result in spurious results as there is no remaining meaningful information after filtering.

We started with *a priori* multiscale entropy parameters based on previous recommendations, although others could have been used. For example, optimization could be used have been used to determine the parameter set that (1) provides most consistent entropy values by providing sufficient pattern matches at *m* and *m*+1 [18, 21] or (2) best distinguishes two population groups [31]. In the present examination of the presence of a consistent difference in age groups, using such approaches to find the parameter set that would provide the best results would be circular logic. Therefore we chose to start from a particular recommendation rather than find those supporting our hypothesis. A comprehensive exploration of the parameter space also would provide a more complete answer [19], but the computational costs are very prohibitive. Our use of *m* = 2–3 and *r* = 0.01–0.35 identified generally consistent patterns across these parameters.

In summary, older adults exhibited lower complexity in proximal leg muscle surface EMG compared to young adults, but higher complexity in the *gastrocnemius* in the 27–270 Hz range. Slower walking corresponded to higher complexity, and thus may allow better neuromuscular function in older adults who walk slower. These changes may be due to neuromuscular noise or adaptations to the requirements of the motor task. Although lower complexity has been observed in many systems with aging, this phenomenon may be obvious in some physiological systems at certain timescales but not others. Studies of motor behavior over a wider range of timescales may be needed to better understand the effects of aging over the motor system.
